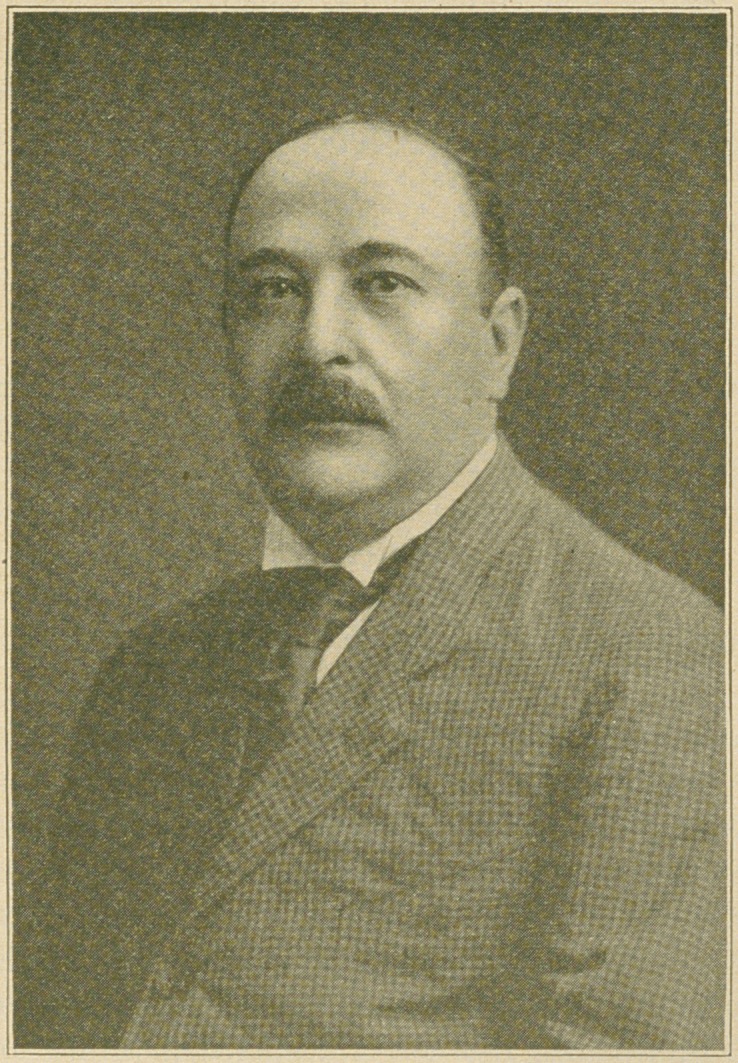# Charles H. Rosenthal, D.D.S.

**Published:** 1914-03-15

**Authors:** 


### OBITUARY.

### Charles H. Rosenthal, D.D.S.

Dr. C. H. Rosenthal was born September 3rd, 1858, in Indianapolis, he died November 19, 1913, at his home in Cincinnati. When one year old his parents moved to Ft. Wayne, Indiana. At the age of seventeen he came to Cincinnati, entered the Ohio College of Dental Surgery and graduated at the head of his class, receiving the gold medal for proficiency. He also studied medicine in this City. He had been practising dentistry in Cincinnati for thirty years. Was prominently connected with the Business Mens Club and the Automobile Club.

Dr. Rosenthal was twice married, his first wife Miss Stella Egan, daughter of the late John Egan, who was for years connected with the Big Four Railroad. His present wife was Miss Ida Thompson sister of Mrs. Bert L. Baldwin. He leaves besides his wife a son John Egan Rosenthal who is a student at the Franklin School of this City and a daughter Mrs. Carelton Fleming of Detroit, Michigan. His father was one of Indianas most prominent physicians. He leaves two brothers, Dr. Maurice Rosenthal a brilliant surgeon known throughout the country and Dr. Milton Rosenthal a well known dentist of Ft. Wayne, three married sisters also survive him. He had a large and lucrative practice in Cincinnati.

Thus does the Grim Reaper take from us our relatives, friends, and genial companions, when comes the time to enter the Great Beyond.

Dr. Rosenthal's many friends among the dentists and dental dealers extend their sympathy to his wife and family in their sad bereavement.

## Figures and Tables

**Figure f1:**